# Supplementary material for: The effect of molnupiravir and nirmatrelvir on SARS-CoV-2 genome diversity in severe models of COVID-19
Source: Microbiol Spectr. 2025 Mar 25;13(5):e01829-24. doi: 10.1128/spectrum.01829-24 (PMC12053996; doi:10.1128/spectrum.01829-24)
Supplement: Supplemental material — Tables S1 and S2; Fig. S1 to S5. [file spectrum.01829-24-s0002.docx]

**SUPPLEMENTARY TABLES**

***Table S1:*** *The number of samples per cohort, sample type and DPI that were included in the sequencing analysis. Samples with less than 90% coverage and poor or mediocre quality control results determined by Nextclade CLI, were excluded from the analysis. ^*^Multiple end point time-points due to humane end-point variation.*

| **Cohort** | **Sample type** | **DPI^*^** | ***n*** |
| --- | --- | --- | --- |
| **Cyclophosphamide and Molnupiravir** | Swab | 1 | 4 |
|  |  | 3 | 4 |
|  |  | 5 | 3 |
|  | Lung Tissue | 7 | 4 |
|  | Nasal Tissue | 7 | 4 |
| **Cyclophosphamide and Nirmatrelvir** | Swab | 1 | 1 |
|  |  | 3 | 2 |
|  |  | 5 | 1 |
|  | Lung Tissue | 4 | 1 |
|  |  | 7 | 2 |
|  | Nasal Tissue | 4 | 1 |
|  |  | 7 | 2 |
| **Cyclophosphamide only** | Swab | 1 | 4 |
|  |  | 3 | 4 |
|  |  | 4 | 1 |
|  |  | 5 | 2 |
|  | Lung Tissue | 4 | 2 |
|  |  | 6 | 2 |
|  | Nasal Tissue | 4 | 1 |
|  |  | 6 | 2 |
| **Cyclophosphamide, Nirmatrelvir and Molnupiravir** | Swab | 1 | 1 |
|  |  | 3 | 2 |
|  | Lung Tissue | 3 | 1 |
|  |  | 7 | 2 |
|  | Nasal Tissue | 3 | 1 |
|  |  | 7 | 3 |
| **Molnupiravir** | Swab | 1 | 4 |
|  |  | 3 | 4 |
|  |  | 5 | 1 |
|  | Lung Tissue | 7 | 3 |
|  | Nasal Tissue | 7 | 1 |
| **Vehicle** | Swab | 1 | 4 |
|  |  | 3 | 4 |
|  |  | 5 | 4 |
|  | Lung Tissue | 6 | 4 |
|  | Nasal Tissue | 6 | 3 |

***Table S2:*** *Unique phenotypic changes in SARS-CoV-2 within the dataset. Input virus amino acids and S: H665Y were excluded.*

| **Amino acid change** | **Occurrence in dataset** | **Tissue type** | **Cohorts** |
| --- | --- | --- | --- |
| **S: D215H** | 2 | Swab | Vehicle,  Cyclophosphamide, Molnupiravir & nirmatrelvir |
| **ORF1a: G2581S** | 1 | Swab | Cyclophosphamide & nirmatrelvir |
| **ORF3a: P42L** | 1 | Swab | Cyclophosphamide & Molnupiravir |
| **ORF3a: Q57H** | 2 | Nasal Tissue  Swab | Cyclophosphamide, Molnupiravir & nirmatrelvir,  Cyclophosphamide only |
| **E: Y42H** | 1 | Swab | Cyclophosphamide & nirmatrelvir |
| **ORF1a: G82D** | 1 | Lung Tissue | Cyclophosphamide, nirmatrelvir and Molnupiravir  Cyclophosphamide, nirmatrelvir and Molnupiravir |
| **S: V47I** |  |  |  |
| **ORF1a: T568I** | 1 | Nasal Tissue | Cyclophosphamide, nirmatrelvir and Molnupiravir |
| **ORF1a: C2160Y** |  |  |  |
| **ORF1b: F312L** |  |  |  |
| **ORF1b: V2515I** |  |  |  |
| **ORF8: F108L** |  |  |  |
| **S: A27T** |  |  |  |
| **ORF1a: F741L** | 1 | Nasal Tissue | Cyclophosphamide & nirmatrelvir |
| **ORF1a: A872T** |  |  |  |
| **ORF7b: S5P** |  |  |  |
| **S: A890V** |  |  |  |
| **S: D985N** |  |  |  |
| **ORF1a: F190L** | 1 | Nasal Tissue | Molnupiravir |
| **ORF1a: G209S** |  |  |  |
| **ORF1a: V2130I** |  |  |  |
| **ORF1b: R1729H** |  |  |  |
| **ORF3a:M1I** |  |  |  |
| **ORF3a: G224S** |  |  |  |
| **S: S247R** |  |  |  |
| **S: V483I** |  |  |  |

**Supplementary Figures**

**Figure S1**


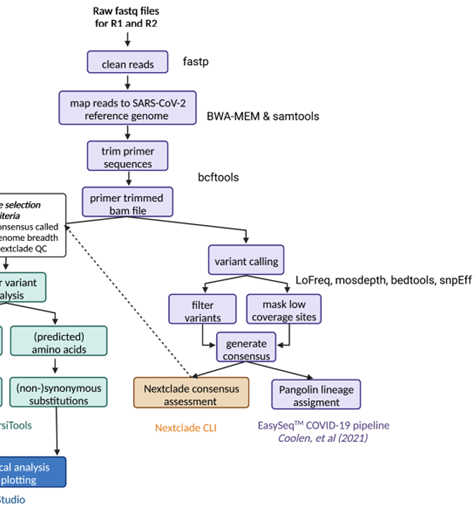


Fig S1: Bioinformatic workflow used for sequencing analysis.


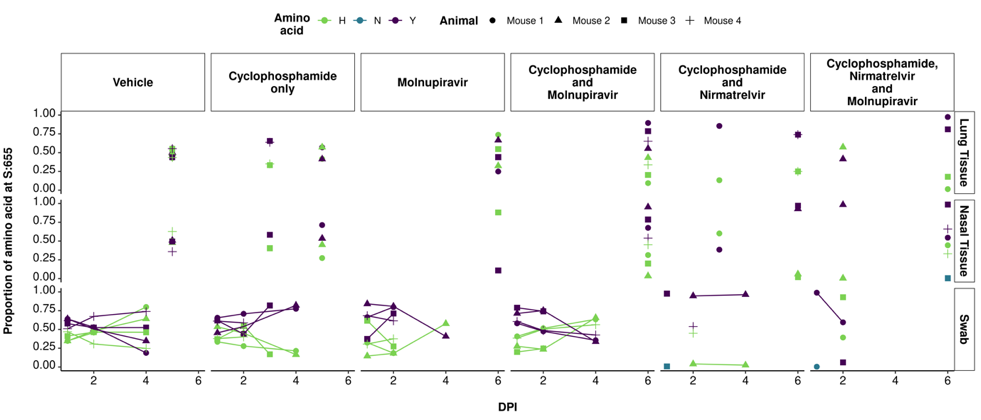


Fig S2: The proportion of amino acid H and Y at position 655 in the Spike protein over time or in lung or nasal tissues. The S: H655Y mutation was observed at the minor variant level if not observed in the consensus level. Each point represents one animal within the cohort.

***
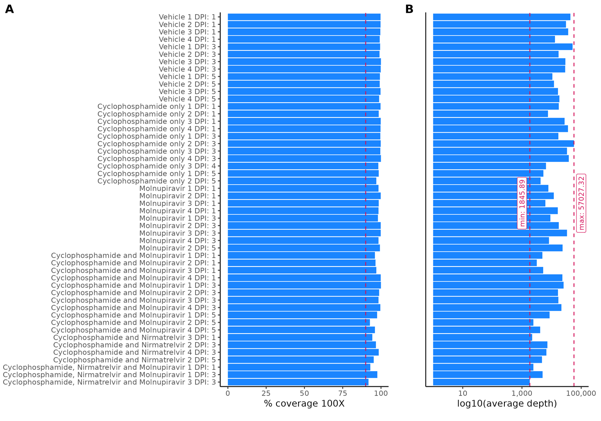
***

Fig S3: The coverage (A) and average depth (B) of the swabs used in the minor variant analysis.


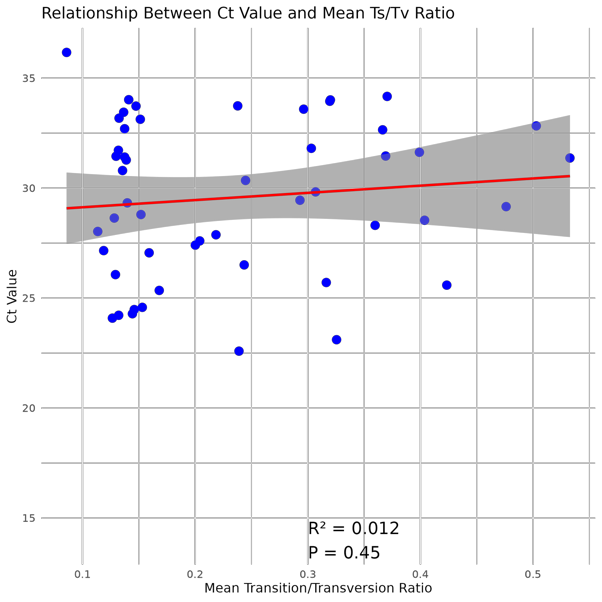


*Fig S4: The scatter plot shows the relationship between Ct values and the mean transition/transversion (Ts/Tv) ratios calculated with the DiversiTools output. Each blue dot represents a swab sample used in the minor variant analysis. The red line indicates the linear regression fit, with the shaded region showing the 95% confidence interval. Statistical analysis revealed no significant correlation between Ct values and Ts/Tv ratios ( R^2 = 0.012 ,  p = 0.45 ), supporting the conclusion that there is no association between the two variables.*

***
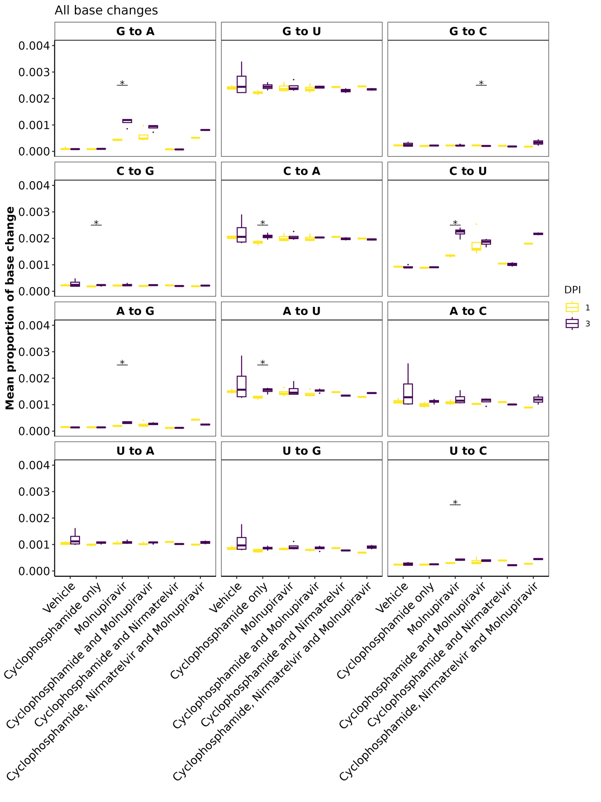
***

*Fig S5: Minor variation for all base changes between day 1 and day 3 post infection. * represents a P value <0.05 (Mann Whitney U test).*
